# Revisiting the MS paradox: automated 2.5D FLAIR segmentation and DTI microstructural profiling reveals dissociation between lesion burden and reference-like intralesional tract profiles

**DOI:** 10.3389/fnhum.2026.1901702

**Published:** 2026-10-20

**Authors:** Edgar A. Toscano-Flores, Mario Alberto Mireles-Ramírez, José de Jesús Guerrero-García, Francisco J. Alvarez-Padilla

**Affiliations:** 1 Departamento de Bioingeniería Translacional, Universidad de Guadalajara, Jalisco, México; 2 Dirección de Enseñanza e Investigación en Salud, Unidad Médica de Alta Especialidad Hospital de Especialidades, Centro Médico Nacional de Occidente, Instituto Mexicano del Seguro Social, Guadalajara, México; 3 Banco Central de Sangre, Unidad Médica de Alta Especialidad Hospital de Especialidades, Centro Médico Nacional de Occidente, Instituto Mexicano del Seguro Social, Guadalajara, México; 4 Departamento de Farmacobiología, Centro Universitario de Ciencias de la Salud, Universidad de Guadalajara, Guadalajara, México

**Keywords:** clinico-radiological paradox, clustering, DTI, MRI, multiple sclerosis, reference-like tract segment, segmentation

## Abstract

**Introduction:**

Magnetic resonance imaging (MRI) reveals macroscopic lesion burden in multiple sclerosis (MS) but correlates poorly with clinical phenotype, a phenomenon known as the clinico-radiological paradox. While diffusion tensor imaging (DTI) metrics assess microstructural damage, standard whole-lesion averaging may obscure microstructurally spared white matter within heterogeneous plaques.

**Methods:**

We propose a fully automated, multimodal pipeline to identify and quantify reference-like intralesional segments (RLIS) within MS lesions (i.e., the cluster exhibiting maximum feature-space similarity to subject-specific, lesion-spared reference tissue). A modified 2.5D U-Net, developed and evaluated on a cohort of 75 MS patients (115 series), performed automated FLAIR lesion segmentation. In an independent cohort of 100 MS subjects, intralesional DTI metrics (FA and MD) were extracted along streamlines reconstructed via Constrained Spherical Deconvolution tractography. Unsupervised clustering algorithms, validated against a subject-specific, lesion-spared reference CST streamlines (internal capsule), identified operationally defined reference-like segments.

**Results:**

The 2.5D U-Net achieved robust segmentation performance (DSC: 0.732, VC: 0.954), exhibited higher nominal metrics in a contextual benchmark comparison (DSC: 0.682, VC: 0.864). Across the clinical cohort, lesion count (11–131) and volume (0.54–57.3 mL) varied widely. Among the evaluated clustering methods, K-Means and Gaussian Mixture Models proved to be the most conservative, identifying median reference-like segment proportions of 26% and 22%, respectively. Crucially, the proportion of reference-like intralesional segments showed no significant monotonic association with macroscopic lesion volume or count.

**Conclusion:**

This automated framework successfully integrates robust deep learning-based lesion segmentation with tract-specific analysis to quantify the proportion of reference-like intralesional segments. The observed dissociation between overall lesion burden and RLIS suggests that this index serves as a promising candidate metric for exploring microstructural features that may contribute to the clinico-radiological paradox.

## 1 Introduction

Multiple sclerosis (MS) is an inflammatory and neurodegenerative demyelinating disease that affects the central nervous system (Hartmann et al., 2023). Magnetic resonance imaging (MRI) enables *in vivo* visualization of lesion activity and burden using sequences such as T2-weighted, fluid-attenuated inversion recovery (FLAIR), and T1-weighted (Hemond and Bakshi, 2018). However, clinical symptoms and MRI parameters such as the number, location, and severity of lesions vary dramatically among patients (Healy et al., 2017). Consequently, the term “clinico-radiological paradox” has arisen to describe the limited correlation between macrostructural MRI measures and the clinical phenotype (Mollison et al., 2017).

Addressing this intralesional heterogeneity, diffusion tensor imaging (DTI) offers improved pathological specificity over conventional MRI for assessing the degree of microstructural damage within individual plaques (Pagani et al., 2007). Mean diffusivity (MD) and fractional anisotropy (FA) are the most widely used DTI metrics. An increase in MD often reflects microstructural changes that facilitate free water diffusion, such as axonal loss, myelin damage, or edema. Conversely, decreased FA indicates a loss of directional diffusion, typically driven by demyelination, inflammatory cell accumulation, or axonal transection (Caranova et al., 2023). Unfortunately, standard radiological assessments that average these DTI metrics across an entire segmented lesion mask dilute this critical information, obscuring the presence of functionally viable white matter.

To perform large-scale, highly localized intralesional analyses of these metrics, highly accurate lesion delineation is required. Manual segmentation is highly challenging, time-consuming, and prone to notoriously high inter- and intra-observer variability. Over the past years, automated deep learning algorithms, particularly U-Net architectures, have yielded excellent results for FLAIR lesion segmentation (Zhang et al., 2019; Wahlig et al., 2023). To balance computational resources and spatial context, 2.5D approaches have emerged, leveraging adjacent slices to provide contextual information comparable to full 3D networks but with significantly lower computational cost (Zhang et al., 2024). Furthermore, the integration of attention gates improves these networks by dynamically assigning higher importance to subtle lesions while suppressing background noise (Hashemi et al., 2022). To further enhance feature propagation and facilitate the training of deeper architectures without performance degradation, residual blocks were incorporated into the encoder-decoder structure (Sarica and Seker, 2022). Finally, a deep supervision mechanism was implemented to mitigate the vanishing gradient problem and force intermediate layers to learn discriminative features at multiple scales (Lin et al., 2021), which is critical for capturing the high variability in MS lesion sizes.

To address the gap between volumetric burden and microstructural integrity, we propose a sequential, fully automated multimodal pipeline. First, we employ an attention-guided 2.5D deep learning model to automate FLAIR lesion segmentation. Subsequently, we reconstruct the localized fiber architecture with tractography and apply an unsupervised clustering algorithm directly onto the intralesional DTI metrics (FA and MD). This integration allows us to dynamically identify reference-like intralesional segment proportion based on their specific microstructural signatures. We hypothesize that this automated framework will reveal a dissociation between lesion burden and reference-like intralesional tract profiles, providing a quantifiable structural descriptor to explore the MS paradox. In this framework, these profiles are operationally defined by intralesional segments exhibiting the highest relative microstructural similarity to an intra-subject, lesion-spared reference.

## 2 Materials and methods

The proposed methodology consists of three main stages: the first involves Volume of Interest (VOI) definition, the second focuses on clustering, and the third centers on statistical analysis (see Figure 1).

**Figure 1:**
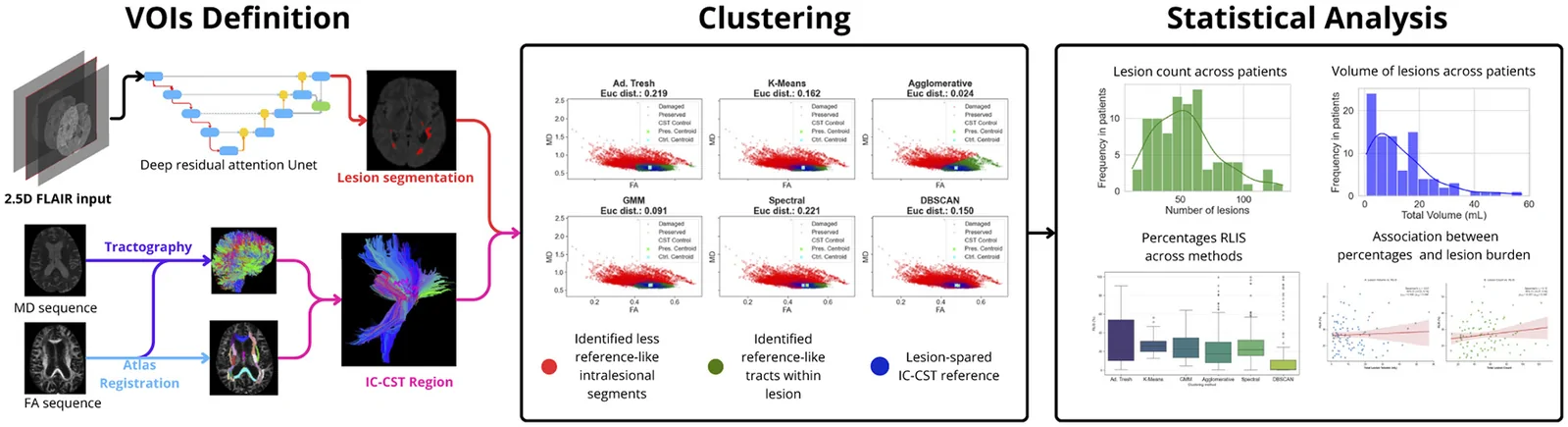
Proposed methodological workflow for automated lesion segmentation, tractography clustering, and downstream statistical analysis. The pipeline is divided into three main operational phases: (1) VOIs definition **(left)**: Multi-sequence MRI data (2.5D FLAIR, MD, and FA sequences) are utilized as inputs. Lesion segmentation is performed using a Deep Residual Attention U-Net, while the Internal Capsule Corticospinal Tract (IC-CST) region is reconstructed through tractography and non-linear atlas registration. (2) Clustering **(center)**: Bivariate microstructural feature spaces (FA vs. MD) are evaluated across six unsupervised classification frameworks: Adaptive Thresholding (Ad. Tresh.), K-Means, Agglomerative Clustering, Gaussian Mixture Models, Spectral Clustering, and DBSCAN. Tract elements inside the lesion are segmented into damaged (red dots) and operationally defined RLIS (green dots), bench-marked against the subject-specific, lesion-spared IC-CST reference baseline (blue circles). Crosses indicate calculated cluster centroids. (3) Statistical analysis **(right)**: Cohort-wide evaluation charting the clinical frequency distributions of lesion counts and total volumes, quantitative comparison of RLIS percentages across the six clustering techniques, and Spearman rank correlation analysis evaluating potential monotonic associations between RLIS percentages and overall lesion burden (total volume and lesion count).

### 2.1 Database and participants

This study was conducted on the secondary analysis of two publicly available and fully de-identified databases, openly accessible through the articles published in Guarnera et al. (2025) and Fiscone et al. (2024). The original data were collected and published under the corresponding ethical approvals of the country of origin. In accordance with Article 17, section I, of the Regulation of the General Health Law on Health Research of Mexico, the present work is classified as research without risk, as it is a retrospective documentary study in which there was no intervention in individuals, and in which it is not possible to identify the persons. Nevertheless, it was verified that the database contains no personal identifiers and does not allow the re-identification of participants. The first dataset was employed for training the U-Net segmentation model and the second for the subsequent DTI metric analysis.

#### 2.1.1 MSLesSeg dataset

For the segmentation training, we used the database published by Guarnera et al. (2025). The dataset contains records from 75 patients (48 female, 27 male), with ages ranging from 18 to 59 years (mean age: 37 ± 10.3 years). Scans were collected at multiple time points: 50 patients had one, 15 had two, 5 had three, and 5 had four, totaling 115 series. From this dataset, FLAIR sequences were extracted along with their corresponding expert-annotated ground truth masks.

#### 2.1.2 Multimodal dataset

The analysis of DTI metrics required a multimodal database containing both FLAIR and DTI sequences. For this purpose, we selected the dataset described by Fiscone et al. (2024), which includes 100 subjects diagnosed with MS and 50 healthy controls. Our analysis focused on the MS cohort, consisting of 58 females and 42 males, aged 23 to 73 years (mean age: 47.6 ± 11.4 years). The repository metadata includes demographic characteristics (age and sex) alongside MRI acquisition parameters, but lacks clinical disability metrics (such as EDSS scores) or cognitive performance evaluations.

### 2.2 Imaging parameters

#### 2.2.1 MSLesSeg dataset

Data were acquired using different scanners with field strengths of 1.5T and 3T. The FLAIR sequences were characterized by a mean spatial resolution of 455.82 × 455.82 ± 314.71 × 314.71 and a mean pixel spacing of 0.75 × 0.75 ± 0.32 × 0.32 mm. The average acquisition parameters were: slice thickness of 2.61 ± 0.62 mm, Repetition Time (TR) of 6,859.95 ± 1,750.69 ms, Echo Time (TE) of 276.31 ± 89.49 ms, Inversion Time (TI) of 2,072.62 ± 340.85 ms, and a flip angle of 90.55° ± 6.23°.

Ground truth masks were generated by one junior rater and validated by two senior experts (a neuroradiologist and a neurologist) specializing in MS.

#### 2.2.2 Multimodal dataset

Imaging data were acquired on a 3T Magnetom Skyra scanner (Siemens Healthineers, Erlangen, Germany) equipped with a 64-channel head/neck receiver coil (Fiscone et al., 2024). For each subject, the acquisition protocol included paired *T*_2_-weighted FLAIR and diffusion-weighted imaging (DWI) sequences. FLAIR images were acquired with a TR of 5, 000ms, TE of 428ms, and TI of 900ms, with a spatial matrix of 256 × 256 × 160 and an isotropic voxel resolution of 1 × 1 × 1 mm^3^.

DWI acquisitions were performed with TR = 4, 300ms and TE = 98ms, utilizing Anterior–Posterior phase encoding along with reversed-phase scans for susceptibility distortion correction, yielding a spatial matrix of 110 × 110 × 88 and an isotropic voxel size of 2 × 2 × 2mm^3^. Although the underlying cohort incorporates both single-shell and multi-shell acquisition schemes, only the 64-direction *b* = 2, 000s/mm^2^ diffusion shell was extracted across all subjects to ensure sequence standardization and technical consistency.

### 2.3 Image preprocessing and tractography

Prior to feature extraction and fiber tracking, the diffusion-weighted volumes were already preprocessed by the dataset curators (Fiscone et al., 2024), incorporating thermal noise reduction (dwidenoise in MRtrix3) as well as susceptibility, eddy-current, and head motion artifact corrections (FSL topup and eddy).

Subsequent processing and fiber tracking were performed using the Dipy library (Diffusion Imaging in Python). To ensure accurate isolation of neural tissue, skull stripping was executed on the native diffusion volumes using the Median Otsu segmentation algorithm. This method applies a median filter to reduce noise before computing the optimal binary threshold, generating a precise brain mask.

To map the white matter architecture in its native space, a standard DTI model was first fitted to the data to calculate scalar maps, specifically FA and MD. Separately, to accurately resolve complex crossing fiber configurations, a Constrained Spherical Deconvolution model was applied. This method deconvolves the observed diffusion signal using an empirically estimated single-fiber response function to compute the fiber Orientation Distribution Function within each voxel, thereby extracting the true principal diffusion directions (peaks) even in heterogeneous regions.

Whole-brain deterministic tractography was then reconstructed natively. Tracking was initiated exclusively from robust white matter regions, defined by an FA > 0.3, placing exactly one seed per voxel. Streamlines were generated utilizing a deterministic Local Tracking algorithm guided by the CSD peaks, with a step size of 0.5 mm and a maximum turning angle of 25 degrees. To prevent incursions into gray matter or cerebrospinal fluid, the tracking was strictly terminated if a streamline entered a voxel with *FA* < 0.20. Finally, a post-processing length filter was applied to retain only streamlines between 30 mm and 250 mm, successfully removing spurious short fibers.

Subsequently, to facilitate the intra-lesional microstructural evaluation, the native datasets were aligned with the structural FLAIR space. A multi-resolution rigid-body transformation framework (spanning three resolution levels: 1,000, 100, and 10 iterations) was optimized using a Mutual Information metric and initialized via center-of-mass alignment. The resulting transformation map was utilized to register the FA and MD scalar maps, as well as to mathematically transform the reconstructed streamlines, into the FLAIR coordinate system. Intensity normalization was subsequently applied to standardize voxel values across subjects.

### 2.4 VOIs definition

Two primary VOIs were established to evaluate microstructural integrity. First, the internal capsule of the corticospinal tract (IC-CST) was identified through atlas-based registration to serve as an automated, pathology-spared reference region. This selection provides a highly reproducible baseline (Wakana et al., 2007) that minimizes contamination from both focal demyelinating plaques (Charil et al., 2007) and diffuse microstructural alterations characteristic of periventricular normal-appearing white matter (Vrenken et al., 2010). Second, an automated deep learning framework was implemented for the voxel-wise segmentation of MS lesions in FLAIR sequences.

#### 2.4.1 IC-CST identification

To accurately localize the IC-CST, we employed a robust, multi-stage registration pipeline to warp the JHU-IMCBM atlas from MNI space to each subject's native diffusion space. The pipeline utilized an optimized sequence consisting of Center of Mass alignment, rigid-body transformation, affine registration, and Symmetric Normalization (SyN) to account for non-linear anatomical variations.

Once the IC-CST mask was mapped to native space, candidate IC-CST streamlines were extracted from the global tractogram. To establish an automated baseline free from plaque pathology, any IC-CST streamline directly intersecting deep-learning-segmented demyelinating lesions was excluded. The remaining lesion-spared IC-CST streamlines served as the subject-specific reference distribution. This procedure ensures that the reference control metrics are precisely aligned with native diffusion geometry while minimizing contamination from overt focal lesion pathology.

#### 2.4.2 Lesion segmentation

Automated lesion segmentation was performed using a Deep Residual Attention 2.5D U-Net, designed to handle the high variability in MS plaque morphology. Our architecture, adapted from the framework by Zhang et al. (2024), leverages a 2.5D approach by processing stacks of three consecutive FLAIR slices to capture local spatial context in the Z-dimension while maintaining computational efficiency.

To enhance feature propagation and facilitate the training of a deeper architecture, we incorporated residual blocks into the encoder–decoder structure. Furthermore, Attention Gates (AGs) were integrated into the skip connections to dynamically weight salient lesion features and suppress irrelevant background noise from healthy tissues. To ensure stable convergence and multi-scale feature learning, the network was trained with Deep Supervision, providing auxiliary gradients to intermediate layers through multi-scale outputs (aux1_out, aux2_out). The resulting architecture is shown in Figure 2.

**Figure 2:**
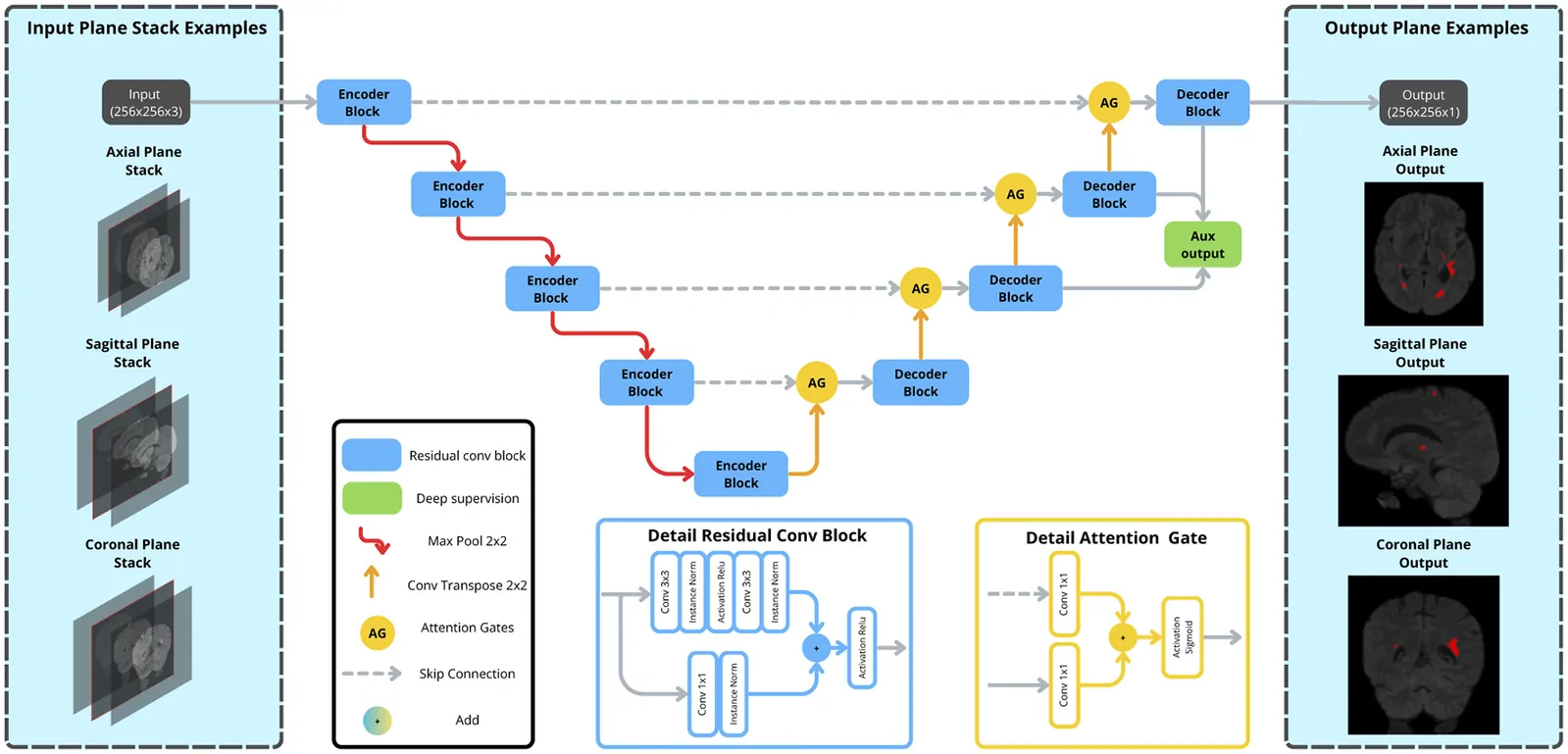
Block diagram of the proposed U-Net architecture. The framework utilizes a 2.5D input configuration (256 × 256 × 3 tensor) to process brain MRI stacks. The backbone is structured upon an encoder-decoder network enhanced with residual convolutional blocks and attention gating mechanisms. As detailed in the bottom insets, the residual blocks employ instance normalization and identity shortcuts, whereas the attention gates utilize 1 × 1 convolutions and sigmoid mappings to dynamically weight skip connection features. Deep supervision is implemented via an auxiliary segmentation branch (Aux output). The final output delivers a single-channel lesion segmentation mask (256 × 256 × 1).

The model was optimized using a Combo Loss (Dice and Focal Tversky Loss) to address the extreme class imbalance between lesions and healthy white matter. To prevent data leakage across the longitudinal scans of the same subject on the MSLesSeg dataset, partitioning was performed at the patient level. Unique patient IDs were randomly assigned to training (64%), validation (16%), and testing (20%).

During inference, we applied a Self-Ensembled Lesion Fusion (SELF) strategy, generating a confidence map from 24 predictions (rotations and flips) and utilizing a double-thresholding approach (τ_1_ for intersection and τ_2_ for connected union) to refine lesion boundaries. The hyperparameters for the SELF strategy were optimized on the validation set using a grid search across the following search space: τ_1_∈[0.5, 0.7] (step size 0.05), τ_2_∈[0.2, 0.4] (step size 0.05), and minimum lesion volume *V*∈{5, 10, 15, 20}.

Finally, model performance was evaluated on the test set using the optimal validation parameters. To account for subject-level variability, 95% confidence intervals (95% CI) were estimated via non-parametric bootstrapping with 1,000 iterations sampled with replacement at the patient level.

### 2.5 Clustering and reference-like segment characterization

To evaluate intralesional microstructural heterogeneity across whole-brain lesions and isolate operationally defined RLIS, features were extracted at the streamline segment level. For each streamline intersecting a plaque, its intralesional segment was assigned the mean FA and MD of the traversed voxels within the binary lesion mask. To account for intra-subject baseline variations and prevent extreme pathological values from distorting feature space, FA and MD were standardized per subject using median–IQR normalization (RobustScaler, Scikit-learn). Fitting the scaler on the combined intra-subject distribution (concatenating lesion-spared IC-CST reference streamlines and intralesional segments) ensured that the normalization domain spanned the full tissue continuum of each patient—from intact white matter to severely degraded lesion cores. Because normalization and distance calculations were performed strictly within each subject, the resulting dimensionless Euclidean distances reflect relative separation within each subject's individual feature space. Euclidean distance was therefore used primarily as an intra-subject criterion to identify the most reference-like cluster, while cross-subject analyses were performed on the resulting RLIS proportion rather than on absolute Euclidean distances.

#### 2.5.1 Adaptive thresholding strategy

We defined a reference range based on the subject-specific lesion-spared IC-CST streamlines statistics, setting threshold boundaries at Mean±β·SD (standard deviation) for both metrics, with a baseline scaling factor of β = 2. Specifically, the FA and MD thresholds were defined as:


$$\begin{array}{ll} {FA}_{t} & ={\bar{FA}}_{CST}-\beta \sigma_{FA_{CST}} \end{array}$$ (1)

$$\begin{array}{ll} {MD}_{t} & ={\bar{MD}}_{CST}+\beta \sigma_{{MD}_{CST}} \end{array}$$ (2)

where Equation 1 represents the threshold for FA, and the Equation 2 for MD. The variables and mathematical definitions used for this stratification are detailed in Table 1. Considering that MS shows decreased FA and increased MD in affected regions, intralesional segments meeting reference-derived thresholds (FA≥FA_*t*_ and MD ≤ MD_*t*_) were operationally classified as reference-like intralesional segments.

**Table 1:** Definition of variables used for the adaptive thresholding method.

Variable	Symbol	Definition
FA threshold	*FA*_*t*_	Lower bound limit for FA based on lesion-spared values.
MD threshold	*MD*_*t*_	Upper bound limit for MD based on lesion-spared values.
Mean FA in IC-CST	${\bar{FA}}_{CST}$	Arithmetic mean of FA values on lesion-spared CST.
Mean MD in IC-CST	${\bar{MD}}_{CST}$	Arithmetic mean of MD values on lesion-spared IC-CST.
STD of FA in IC-CST	σ_*FA*_	Dispersion of FA values on lesion-spared IC-CST.
STD of MD in IC-CST	σ_*MD*_	Dispersion of MD values on lesion-spared IC-CST.

To evaluate the impact of this threshold selection and validate our baseline choice (β = 2), we conducted a sensitivity analysis by varying β∈{0.5, 1.0, 1.5, 2.0}. At each level, we evaluated the percentage of RLIS and the Euclidean distance between the RLIS centroid and the control centroid, the threshold was selected as an explicit heuristic trade-off to maintain adequate intralesional sample volume for stable clustering while preserving microstructural specificity relative to the pathology-spared baseline (see Section 3).

#### 2.5.2 Unsupervised machine learning models

To capture the complex, non-linear microstructural heterogeneity of lesion tissue, we implemented five unsupervised machine learning algorithms: K-Means, Gaussian Mixture Model (GMM), Agglomerative Clustering, Spectral Clustering, and DBSCAN (Patel and Singh Kushwaha, 2020; Tokuda et al., 2022; Jia et al., 2013; Doad and Bartere, 2013).

Considering the high inter-subject variability inherent in DTI metrics, hyperparameter optimization was performed individually for each patient. A grid search was executed per subject to dynamically select the parameters that yielded optimal cluster separation. For models requiring an *a priori* number of clusters, *k* was set to 4 as a domain-informed heuristic reflecting the expected microstructural combinations in the two-dimensional FA-MD feature space. Crucially, these models were not forced into fixed geometric quadrants; instead, after fitting, the most reference-like cluster was operationally identified as the cluster exhibiting the minimum Euclidean distance between its centroid and the pathology-spared reference centroid in scaled space.

#### 2.5.3 Reference-like intralesional segment proportion

For all models, the cluster or subset whose scaled microstructural profile minimized the distance to the subject's reference distribution was identified as the reference-like segment set. The final RLIS percentage was operationally defined as the percentage of intralesional segments exhibiting maximum feature-space similarity relative to the intra-subject, lesion-spared IC-CST baseline (Equation 3):


$$\begin{array}{l} P_{\text{RLIS}}=\left(\frac{N_{\text{RLIS}}}{N_{\text{total\_intralesion\_segments}}}\right)\times 100 \end{array}$$ (3)

### 2.6 Statistical analysis

Statistical analyses were conducted to evaluate the relationship between macrostructural disease burden and microstructural similarity (RLIS proportion). To achieve this, three primary patient-level metrics were computed: total lesion volume, total lesion count, and the percentage of RLIS. Given that MRI-derived lesion volumes and DTI metrics in MS frequently exhibit skewed, non-normal distributions, non-parametric approaches are considered the standard in the literature (Lapucci et al., 2022; Li et al., 2014). Consequently, to test our primary hypothesis regarding the structural basis of the clinico-radiological paradox, Spearman's rank correlation coefficients (*r*_*s*_) with 95% bootstrap confidence intervals (2,000 iterations) were calculated between macrostructural burden (lesion volume and count) and intralesional microstructural similarity across all clustering models. To control the False Discovery Rate, *p*-values were adjusted using the Benjamini-Hochberg procedure (*p*_adj_) applied across the complete family of 12 hypothesis tests (6 clustering models × 2 macrostructural metrics: lesion volume and lesion count). Statistical significance was set at *p*_adj_ < 0.05.

## 3 Results

### 3.1 VOIs extraction

The successful identification of both anatomical and pathological VOIs was achieved for all subjects in the cohort. The multi-stage registration pipeline, utilizing Symmetric Normalization, provided a precise alignment of the JHU-IMCBM atlas to the patient's native diffusion space. This allowed for the reliable extraction of the IC-CST. Visual inspection confirmed that the warped atlas masks strictly adhered to the anatomical boundaries of the CST across all planes (Figure 3).

**Figure 3:**
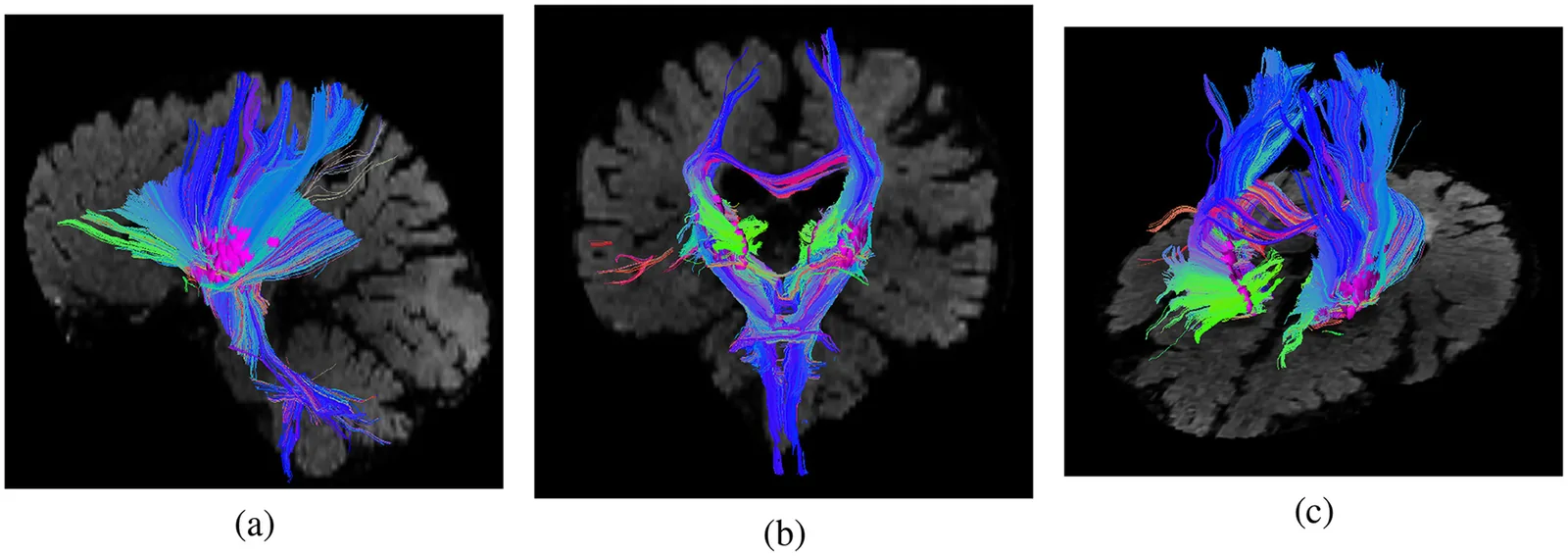
Diffusion tensor tractography of the CST. Directional color-coded maps illustrate the orientation of white matter tracts based on a standard 3D coordinate system: red indicates left-right orientation, green represents anterior-posterior orientation, and blue denotes superior-inferior orientation. Mixed hues (e.g., orange/yellow, magenta, and cyan) reflect the additive combination of vectors for tracts with oblique trajectories. IC-CST shown in purple across three anatomical planes: **(a)** sagittal, **(b)** coronal, and **(c)** angled axial views.

Simultaneously, the Deep Residual Attention 2.5D U-Net effectively delineated the MS lesions. The proposed architecture delineated both large confluent plaques and small hyperintense lesions, subtle hyperintensities that are often overlooked by standard architectures. As shown in Figure 4, the automated segmentation provided a high degree of spatial overlap with the ground truth. These two VOIs (the subject-specific lesion-spared IC-CST reference and the lesion-affected segments) formed the basis for the subsequent voxel-wise microstructural analysis of the DTI metrics (MD and FA).

**Figure 4:**
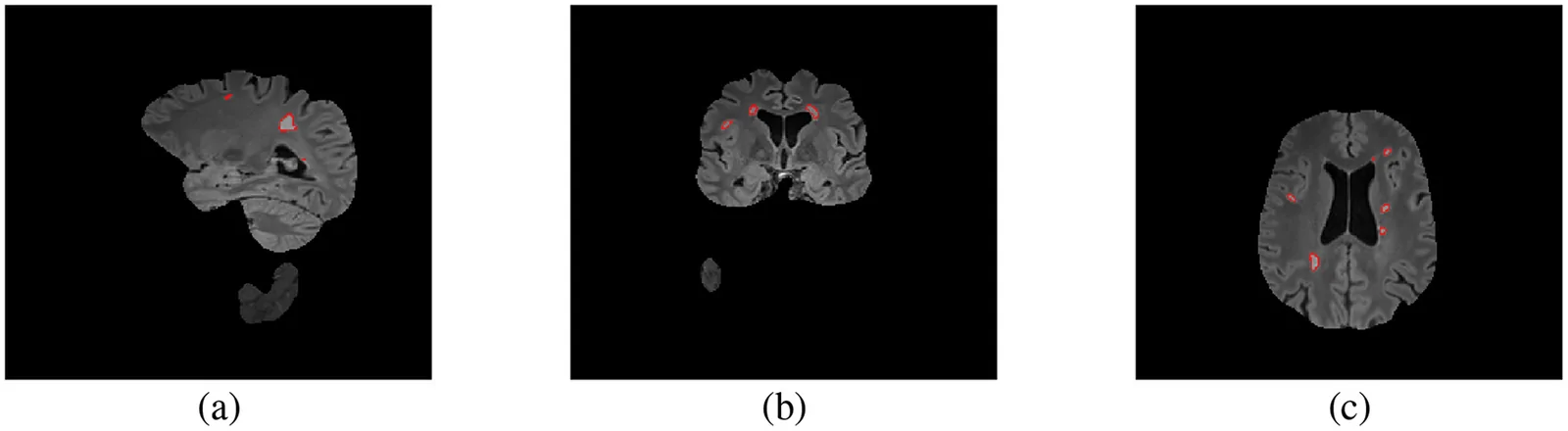
Representative automated lesion segmentation results across orthogonal anatomical planes. The panels display structural brain MRI slices overlaid with red contours delineating the predicted lesion boundaries generated by the proposed U-Net framework. The qualitative performance is showcased across three standard views: **(a)** sagittal, **(b)** coronal, and **(c)** axial. Lesion boundaries are rendered exclusively as line contours (red outlines) to preserve the visibility of the underlying tissue characteristics, signal intensities, and focal pathology within the segmented regions.

### 3.2 Segmentation performance

The performance of the proposed model was evaluated against the benchmark results reported by (Zhang et al., 2024). Through an exhaustive grid search, the optimal inference parameters for the Self-Ensembled Lesion Fusion (SELF) strategy were identified as τ_1_ :0.5, τ_2_:0.4, and a minimum voxel threshold of 15. Under this configuration, as we show in Table 2 our model achieved a higher Dice Similarity Coefficient (DSC) of 0.732, compared to the 0.682 reported in the original study. The largest nominal differences were observed in detection sensitivity: the Lesion-wise True Positive Rate (LTPR) increased from 0.542 to 0.831, and the Voxel-wise TPR rose from 0.613 to 0.781. Furthermore, the Volume Correlation (VC) reached 0.954, indicating an exceptional reliability in estimating total lesion load compared to the 0.864 achieved by the previous method.

**Table 2:** Performance comparison metrics between the original study by Zhang et al. (2024) and the proposed model.

Metric	Zhang et al. (2024)	Proposed model mean [95% CI]
DSC	0.682	**0.732 [0.689–0.777]**
TPR	0.613	**0.781 [0.726–0.843]**
LTPR	0.542	**0.831 [0.778–0.879]**
VC	0.864	**0.954 [0.914–0.995]**
PPV	**0.855**	0.710 [0.644 - 0.780]
LFPR	**0.121**	0.285 [0.213–0.361]

Bold values indicate the best nominal score for each metric. Values from Zhang et al. (2024) are presented as a descriptive reference benchmark.

### 3.3 Lesion burden

The descriptive analysis of the lesion burden revealed wide heterogeneity among the patients in the cohort. The number of lesions per subject ranged from 11 to 131, with a distribution concentrated primarily between 30 and 60 lesions, as shown in the histogram in Figure 5.

**Figure 5:**
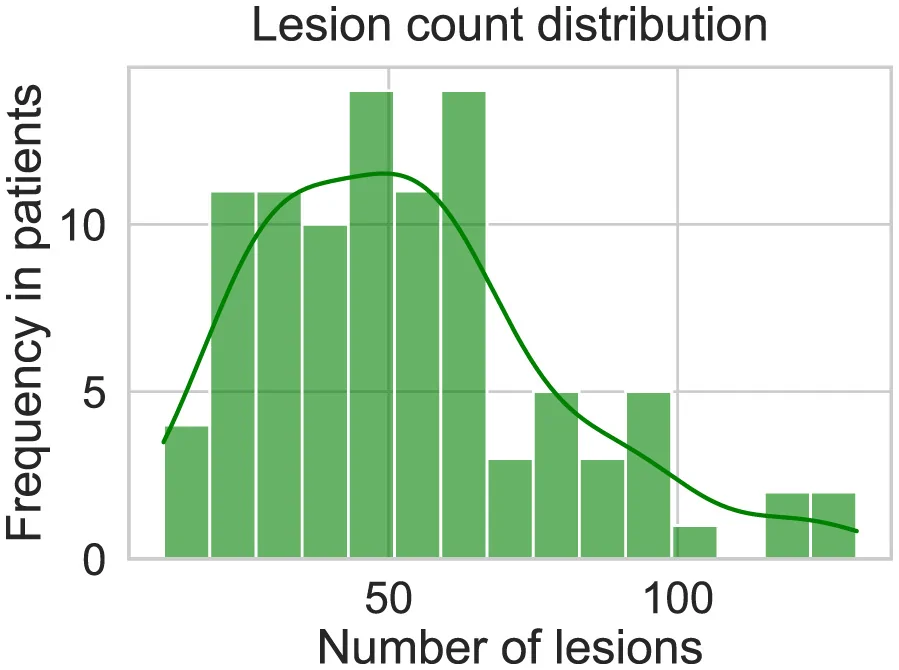
Distribution of segmented lesion counts within the patient cohort. The histogram displays the stratification of lesion numbers per subject, overlaid with a kernel density estimation (KDE) curve to model the underlying continuous distribution. The cohort exhibits a distinct peak, with the majority of patients clustering between 30 and 60 discrete lesions.

Regarding the lesion volume, the distribution exhibited a pronounced right-skewed morphology (see Figure 6), indicating that most subjects presented low to moderate volumes, while a minority of “outlier” patients exhibited extensive lesion loads. Specifically, total lesion volumes varied from a minimum of 0.54 mL to a maximum of 57.3 mL. This variability in both count and volume is essential for evaluating the robustness of the subsequent clustering algorithms under different pathological conditions.

**Figure 6:**
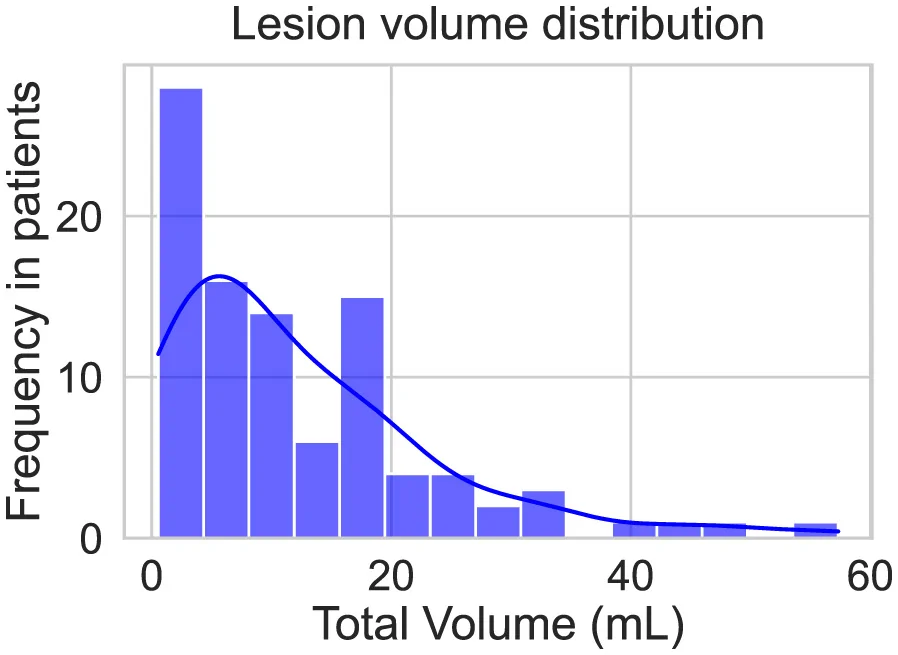
Distribution of total lesion volume across the MS cohort. The histogram and corresponding kernel density estimation (KDE) curve map the total lesion volume in milliliters (mL). The data demonstrates a highly right-skewed (positively skewed) distribution, indicating that while a few outlier cases present severe lesion loads exceeding 40 mL, the clinical baseline for the majority of patients remains below 20 mL.

### 3.4 Tract Clustering

An example of the clustering methods is shown in Figure 7. The estimation of identified RLIS showed a great dependency on the choice of the clustering algorithm (Figure 8). Feature-space models exhibited the highest degree of distribution stability, with K-Means and GMM yielding median RLIS percentages of 26% (IQR: 20%–31%) and 22% (IQR: 13%–35%), respectively. In Figures 9, 10 are shown examples of the created clusters with these algorithms in multiples patients.

**Figure 7:**
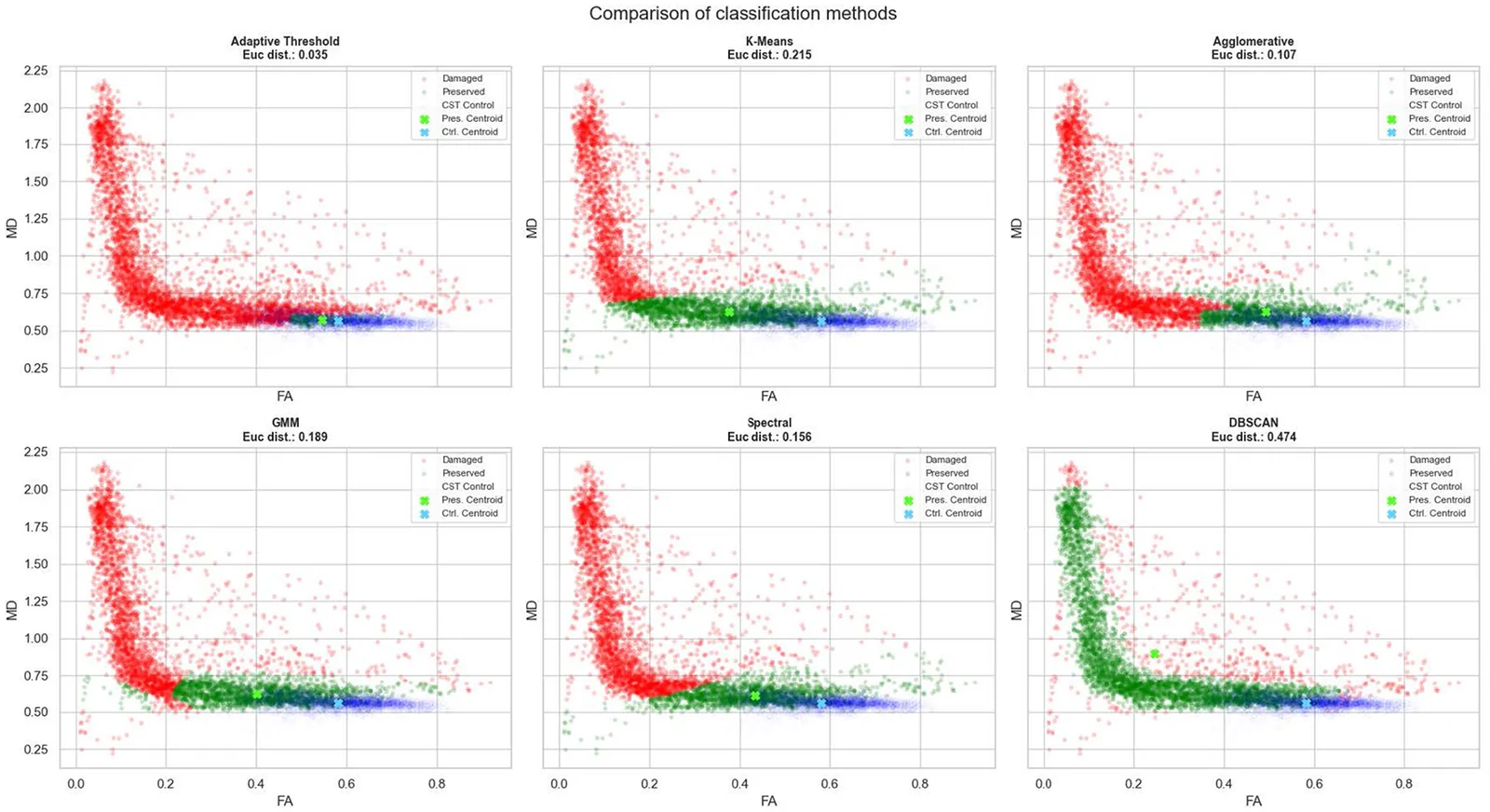
Comparative analysis of unsupervised clustering methods in a single representative patient. The panels illustrate the distribution of Fractional Anisotropy (FA, *x*-axis) versus Mean Diffusivity (MD, *y*-axis) evaluated using six different algorithmic frameworks: Adaptive Thresholding (Ad. Tresh), K-Means, Agglomerative Clustering, GMM, Spectral Clustering, and DBSCAN. Individual data points within the lesion zone are segmented into less RLIS (red dots, labeled as damaged in the legend) and RLIS (green dots, labeled as preserved in the legend), shown relative to the reference Internal Capsule of the Corticospinal Tract (IC-CST) reference profile (blue empty circles, labeled as cst control in the legend). Cross markers ('*X*') denote the calculated centroids for the Reference-like cluster (green, labeled as Pres. centroid) and the baseline reference centroid (cyan, labeled as Ctrl. centroid). The Euclidean distance (Euc dist.) reported above each subplot quantifies the spatial divergence between both centroids in the normalized feature space, highlighting how different clustering geometries affect tissue boundary definition on identical patient data.

**Figure 8:**
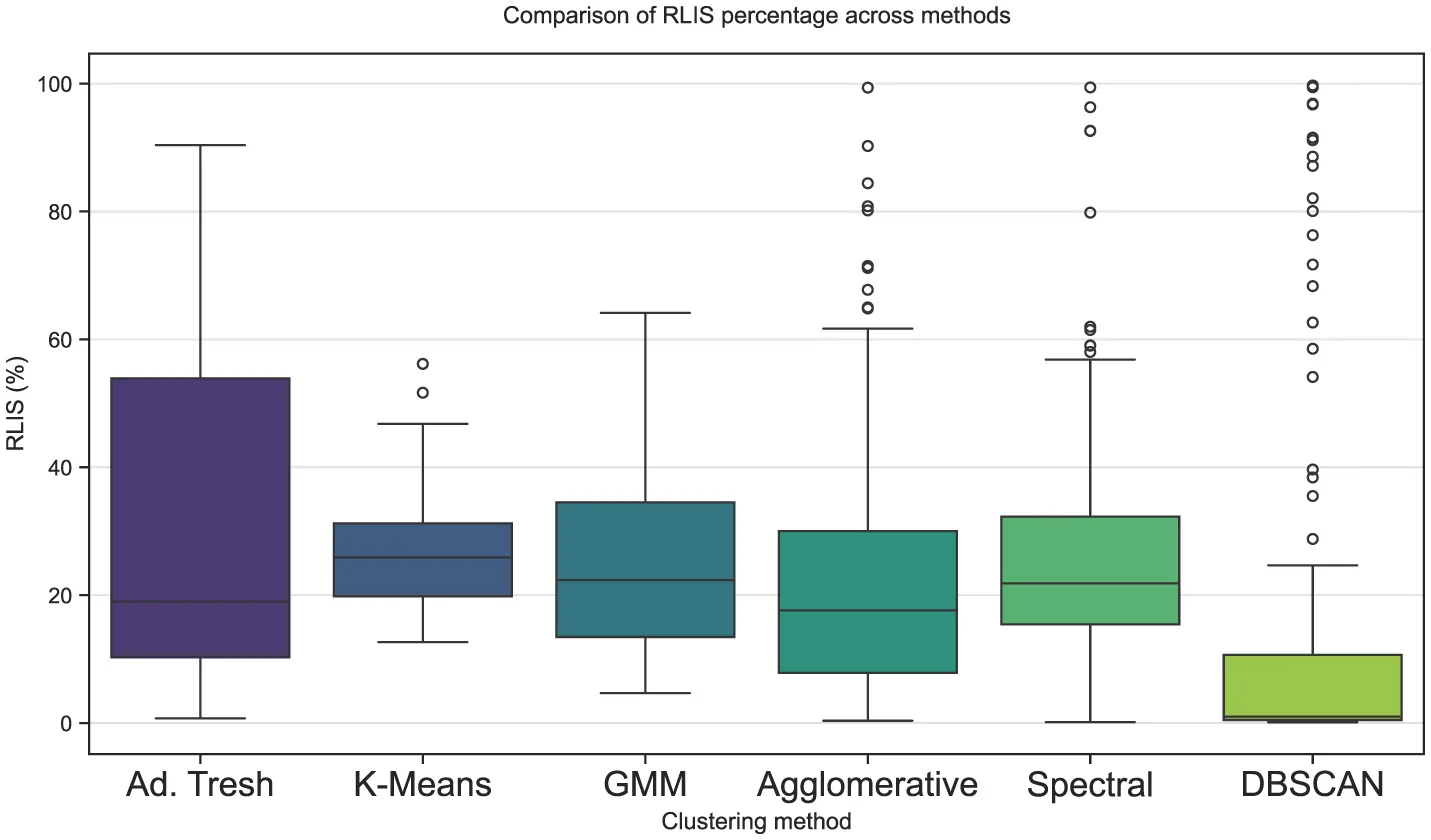
Algorithmic variability RLIS assessment across the evaluated clustering techniques. The distribution of reference-like segment percentages across whole-brain lesions (evaluated relative to the subject-specific, lesion-spared IC-CST reference baseline) is contrasted for Adaptive Thresholding (Ad. Tresh), K-Means, Gaussian Mixture Models (GMM), Agglomerative Clustering, Spectral Clustering, and DBSCAN. Bounding boxes define the 25th and 75th percentiles (interquartile range), internal horizontal lines mark median values, and whiskers indicate the non-outlier range, with hollow circles denoting individual patient outliers. The marked differences in dispersion, ranging from the conservative, tightly bounded estimates of K-Means and GMM to the wide outliers in DBSCAN, demonstrate the mathematical impact of model selection on downstream intralesional microstructural metrics.

**Figure 9:**
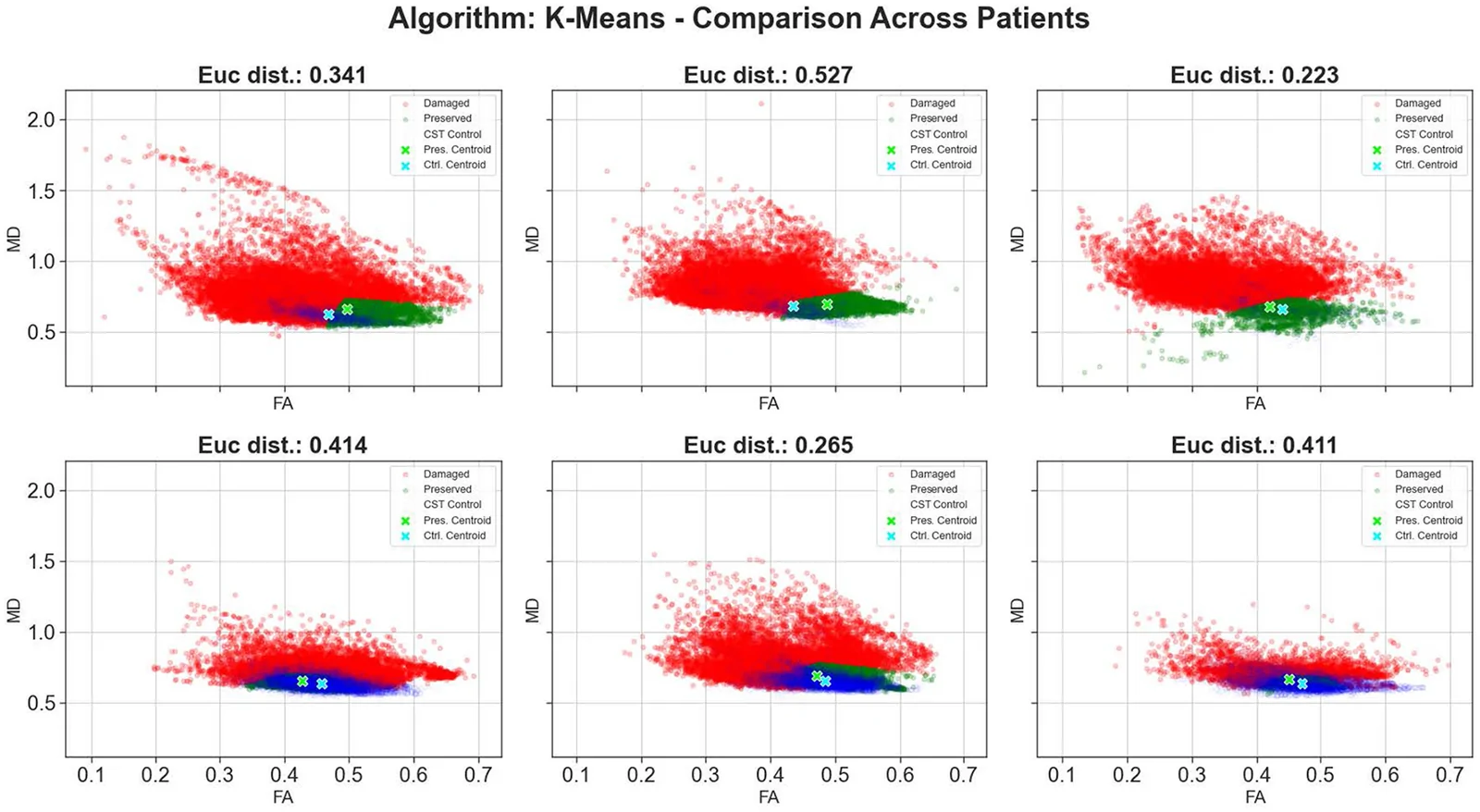
Cross-patient reproducibility of K-Means clustering. The panels display the clustering results for six distinct patient cases using the K-Means algorithm. Color-coding, cluster symbols, and axis metrics follow the exact conventions established in Figure 7. The reported Euclidean distance (Euc. dist.) tracks patient-specific deviations from the lesion-spared reference baseline.

**Figure 10:**
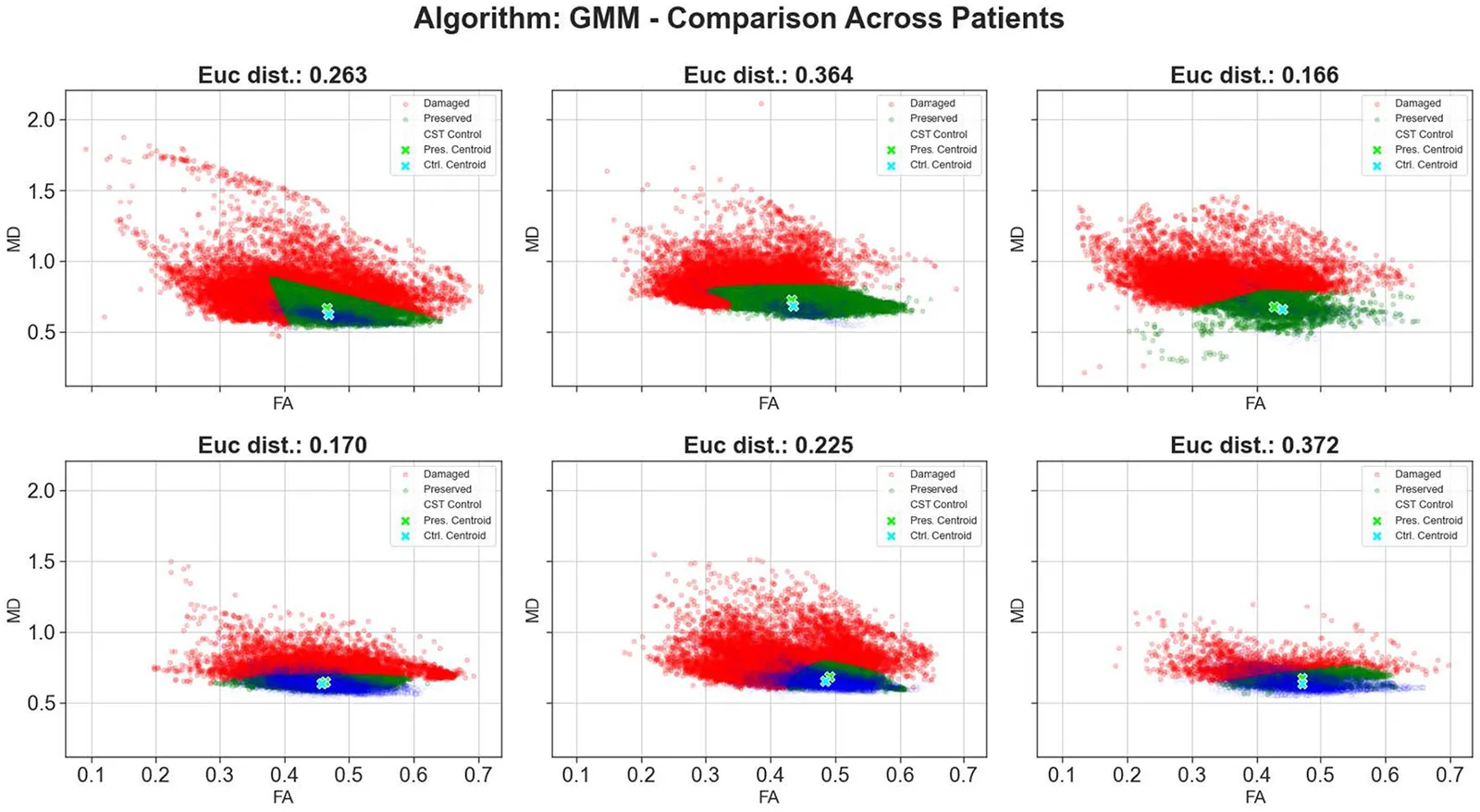
Cross-patient reproducibility of GMM clustering. Visual conventions and parameters are identical to those in Figures 7, 9, mapping the GMM clustering performance across six different subjects.

In contrast, DBSCAN and Agglomerative clustering demonstrated high inter-subject variability, with preservation estimates spanning from near 0% to 100%. The adaptive thresholding method occupied a balanced intermediate position, yielding a mean preservation percentage of 32.34% at our baseline threshold of β = 2. This baseline threshold (β = 2) was established through a sensitivity analysis evaluating parameter values β∈{0.5, 1.0, 1.5, 2.0} (Table 3). As expected, the percentage of RLIS increased monotonically with threshold width, ranging from 15.93% at β = 0.5 to 32.34% at β = 2.0, reflecting broader spatial inclusion as the cutoff relaxed. The mean Euclidean distance between the RLIS centroid and the control centroid was minimized at β = 1.5 (0.480) and increased at narrower thresholds (β = 0.5). The elevation at β = 0.5 likely stems from sample instability due to overly restrictive filtering. Ultimately, β = 1.5 retained only 25.55% of intralesional segments, whereas β = 2 preserved 32.34% of segments with a comparable distance metric (0.492). Thus, β = 2 was retained as a heuristic multi-objective compromise, maximizing statistical power and sample stability for downstream clustering in this dataset.

**Table 3:** Sensitivity analysis across standard deviation thresholds (β).

Threshold (β)	Mean RLIS (%)	Euclidean distance
0.5	15.93	0.680
1.0	20.10	0.529
1.5	25.55	0.480
2.0	32.34	0.492

Crucially, feature-space models were not constrained by fixed geometric boundaries; instead, after fitting, the most reference-like cluster was operationally identified as the one exhibiting the minimum Euclidean distance to the subject-specific, lesion-spared reference centroid in scaled feature space. As shown in Figure 11, the resulting Euclidean distances remained consistently low across subjects (mean < 0.8 in the scaled space), indicating that the operationally selected clusters generally remained close to the subject-specific reference.

**Figure 11:**
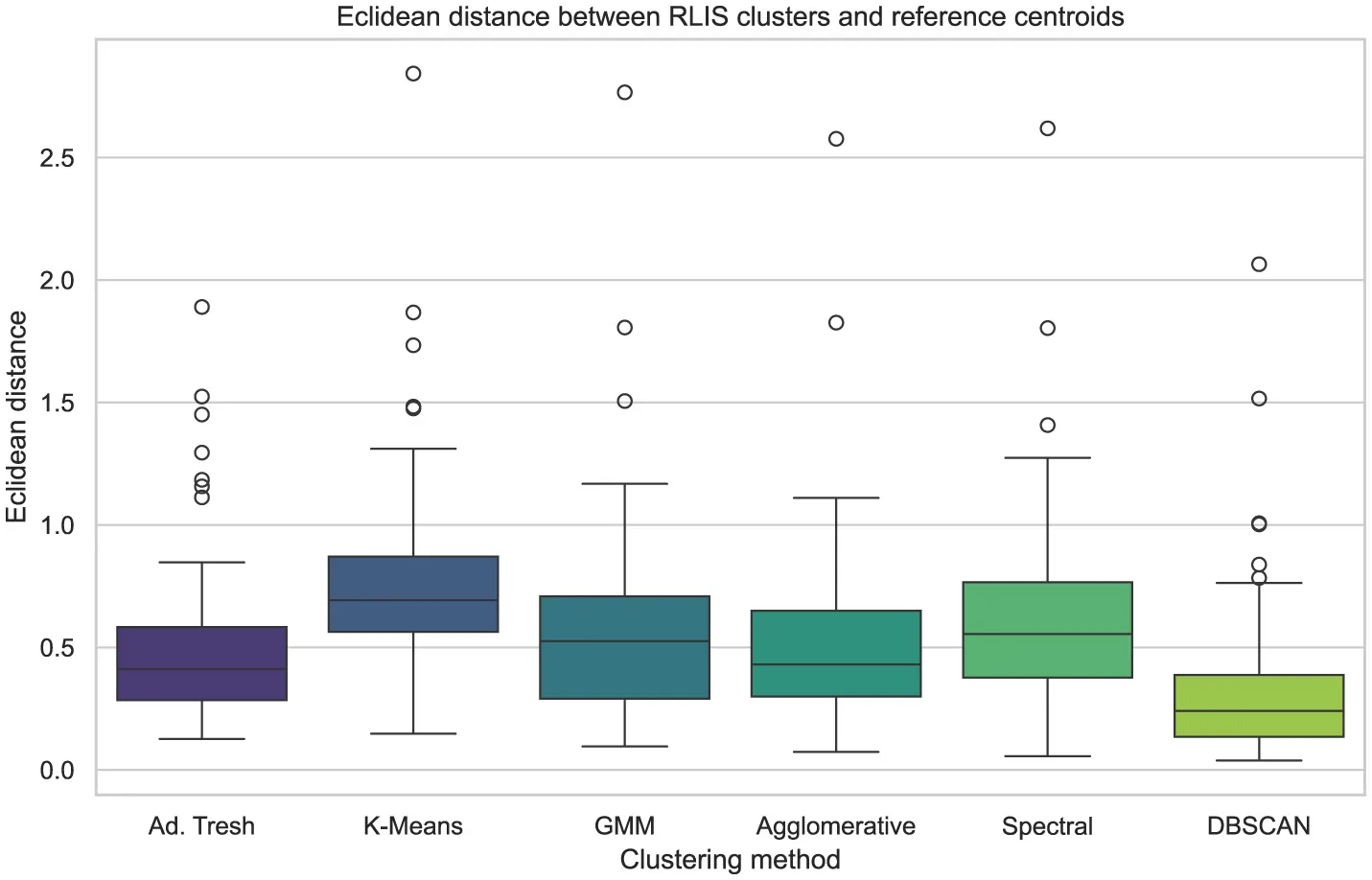
Algorithmic variability in feature-space proximity to subject-specific lesion-spared references. The distribution of Euclidean distances between the most reference-like corticospinal tract clusters and intra-subject reference centroids is contrasted for Ad. Tresh, K-Means, GMM, Agglomerative, Spectral, and DBSCAN methods. Bounding boxes define the 25th and 75th percentiles, internal horizontal markers represent medians, and whiskers indicate the non-outlier range. Hollow circles highlight individual outlier patient cases. The notable differences in proximity, ranging from the lower median distances in Agglomerative and Adaptive Thresholding to the elevated distance baseline in Spectral and K-Means, demonstrate the mathematical trade-off between volume selection stability and microstructural feature fidelity to reference tissue.

Notably, the Spectral algorithm exhibited a bimodal-like distribution with multiple high-value outliers, frequently identifying 100% of the tracts as RLIS in several cases, suggesting a distinct sensitivity to the microstructural features of the sample.

### 3.5 Correlation analysis

Spearman rank correlation analysis showed no statistically significant monotonic associations between the percentage of RLIS and macrostructural lesion burden metrics across all evaluated algorithms (Figure 12, Table 4).

**Figure 12:**
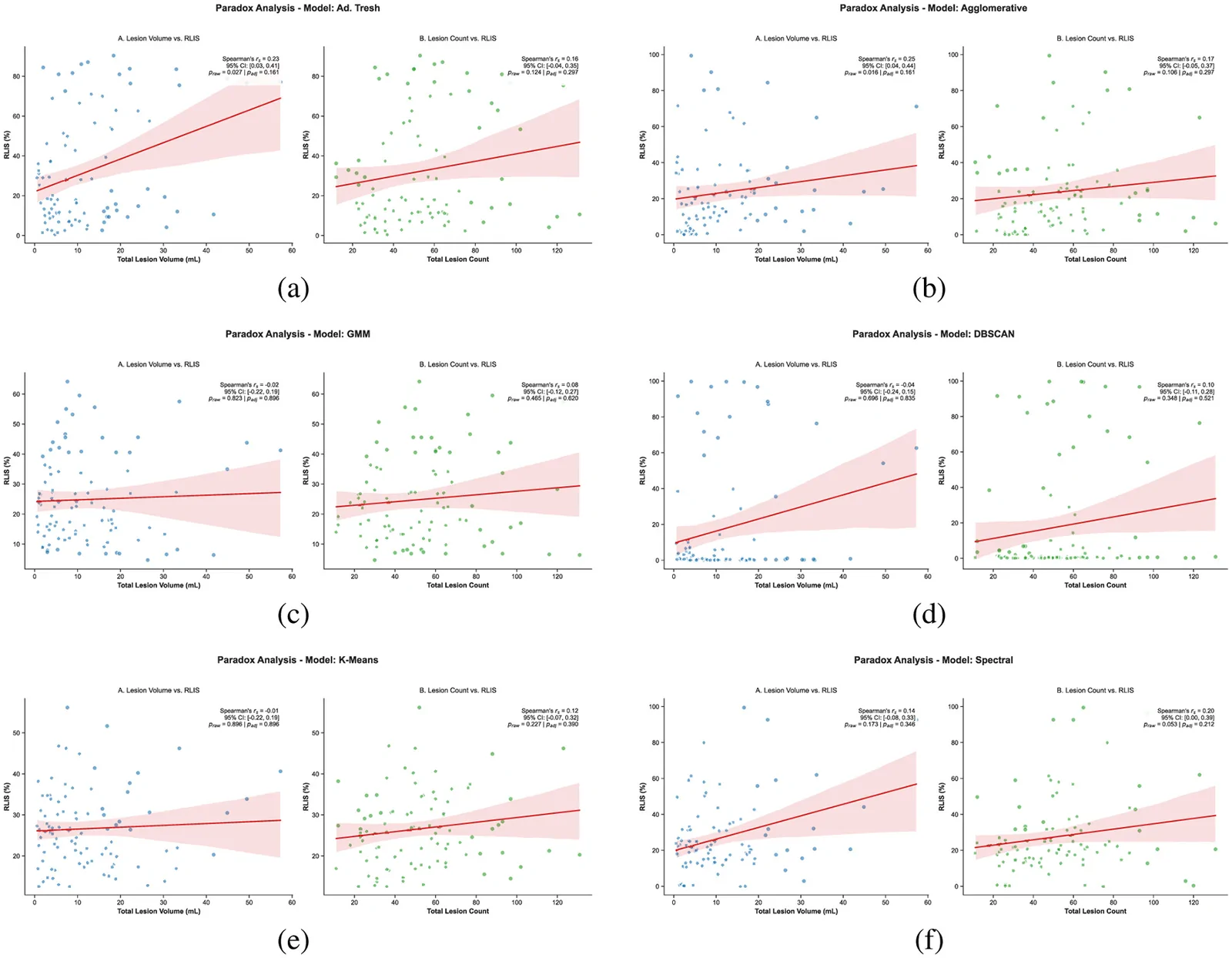
Correlation between macrostructural lesion burden and the percentage of RLIS across different isolation methods. The subfigures display the results obtained using: **(a)** Adaptive Thresholding, **(b)** Agglomerative Clustering, **(c)** GMM, **(d)** DBSCAN, **(e)** K-Means, and **(f)** Spectral Clustering.

**Table 4:** Spearman rank correlations (*r*_*s*_) with 95% bootstrap confidence intervals and FDR-adjusted *p*-values between microstructural RLIS percentages and macrostructural lesion metrics across analytical methods.

	Total lesion volume (mL)		Total lesion count	
Model	*r*_*s*_ [95% CI]	*p*_*adj*_	*r*_*s*_ [95% CI]	*p*_*adj*_
Spectral	-0.14 [–0.08, 0.33]	0.346	0.20 [-0.00, 0.39]	0.212
Agglomerative	-0.25 [-0.04, 0.44]	0.161	0.17 [–0.05, 0.37]	0.297
Adaptive Thresholding	-0.23 [-0.03, 0.41]	0.161	0.16 [–0.04, 0.35]	0.297
GMM	–0.02 [–0.22, 0.19]	0.896	0.08 [–0.12, 0.27]	0.620
DBSCAN	–0.04 [–0.24, 0.15]	0.835	0.10 [–0.11, 0.28]	0.521
K-Means	–0.01 [–0.22, 0.19]	0.896	0.12 [–0.07, 0.32]	0.390

CI, Confidence Interval (calculated via 2,000 bootstrap iterations); FDR, False Discovery Rate (Benjamini–Hochberg correction); GMM, Gaussian Mixture Model; All *p*_adj_>0.05.

Following Benjamini–Hochberg FDR adjustment, no clustering model demonstrated a statistically significant correlation with either total lesion volume (*p*_adj_≥0.161) or lesion count (*p*_adj_≥0.212). Overall, effect sizes across all evaluated methods remained weak to negligible, with *r*_*s*_ point estimates ranging from −0.04 to 0.25.

Feature-space models (K-Means, GMM, and DBSCAN) showed virtually no monotonic association with total lesion volume (*r*_*s*_ = −0.01, −0.02, and −0.04, respectively; all *p*_adj_≥0.835). Conversely, Agglomerative clustering (*r*_*s*_ = 0.25, 95% CI [0.04, 0.44]) and Adaptive Thresholding (*r*_*s*_ = 0.23, 95% CI [0.03, 0.41]) exhibited weak positive unadjusted trends with lesion volume; however, neither remained statistically significant after multiple comparison correction (both *p*_adj_ = 0.161).

Similarly, associations with total lesion count were weak across all models (*r*_*s*_ ≤ 0.20, *p*_adj_≥0.212), with Spectral clustering exhibiting the highest nominal point estimate (*r*_*s*_ = 0.20, 95% CI [0.00, 0.39], *p*_adj_ = 0.212). These findings indicate that the proposed tract-preservation metric showed weak or negligible monotonic associations with conventional macrostructural lesion burden in this cohort.

## 4 Discussion

The results of this study demonstrate the effectiveness of a multimodal pipeline for MS lesion segmentation and the subsequent identification of RLIS architecture within those lesions. The primary finding is the lack of significant monotonic association between the RLIS proportion and traditional global lesion burden metrics (volume and count). This lack of strong association suggests that our metric may capture microstructural features of MS pathology that are not reflected in conventional macrostructural radiological measures.

When contextually benchmarked against reported values in the literature (Zhang et al., 2024), the proposed model exhibited a higher nominal detection sensitivity, characterized by a LTPR of 0.831 [95% CI: 0.778–0.879] compared to the published benchmark of 0.542, and a TPR of 0.781 [95% CI: 0.726–0.843] relative to 0.613. We hypothesize that this enhanced sensitivity may be facilitated by the integration of Attention Gates and Deep Supervision, which emphasize subtle, small-scale pathological features and promote multi-scale feature learning, potentially facilitating their detection. However, this increased detection capability entails a measurable trade-off in precision, with the PPV decreasing to 0.71 and the LFPR rising to 0.285. In clinical neuroimaging, such false positives can potentially complicate the interpretation of lesion counts or bias subsequent microstructural analyses, such as DTI. Despite the higher frequency of individual false detections, the model reached an exceptional VC of 0.954 [95% CI: 0.914–0.995] relative to the published baseline of 0.864. This high correlation suggests that the additional false positives are volumetrically small and do not substantially distort the overall estimation of the lesion burden. To further stabilize these results, the implementation of a 15-voxel minimum threshold served as a critical post-processing filter, effectively suppressing sub-clinical noise while preserving the delineation of true MS plaques. Consequently, this approach provides a robust framework for capturing subtle pathological changes while maintaining a highly reliable global volumetric assessment.

The choice of clustering algorithm substantially influenced both the operational volume estimation and the feature-space proximity of the identified reference-like segments. Evaluating cluster distributions alongside centroid Euclidean distances reveals distinct operational trade-offs across architectural families. Feature-space models, specifically K-Means and GMM, emerged as the most volume-stable architectures, exhibiting bounded interquartile ranges with median preservation percentages centered at 26% and 22%, respectively. However, microstructural distance analysis demonstrated that GMM exhibited a lower median feature-space distance to the subject-specific reference (median Euclidean distance of 0.52), whereas K-Means exhibited a higher baseline distance offset (median of 0.69) due to its rigid hyper-spherical clustering constraints. In contrast, non-parametric models exhibited severe structural or operational degeneration. Density-based clustering (DBSCAN) collapsed to near 0% volume preservation for most subjects (median ≈0%), yielding an artificially low median Euclidean distance (0.24) driven solely by ultra-conservative residual clusters alongside extreme upper outliers reaching up to 100% preservation. Hierarchical (Agglomerative) and graph-based (Spectral) models maintained moderate volume medians (18% and 22%) and low-to-moderate distance baselines (medians of 0.43 and 0.55), but frequently produced unconstrained classifications of up to 100% preservation. In Spectral clustering, this artifact stems from its reliance on a global similarity graph, where subtle diffusion gradient variations may promote affinity oversmoothing and reduced cluster separability, failing to resolve internal tissue boundaries. Adaptive Thresholding presented marked inter-subject volume variance (0% to 83%) despite a low median feature distance (0.41). Consequently, graph-based (Spectral), density-based (DBSCAN), and hierarchical (Agglomerative) algorithms appear ill-suited for this application, whereas feature-space models (K-Means, GMM) produced comparatively more stable estimates.

The absence of statistically significant monotonic correlations across all six evaluated models (following FDR adjustment) is consistent with the hypothesis that the percentage of RLIS offers complementary structural information to global macrostructural metrics. These findings indicate that local intralesional tract “preservation” does not strongly co-vary with overall lesion volume or count. Notably, feature-space models such as K-Means (*r*_*s*_ = −0.01, *p*_adj_ = 0.896) and GMM (*r*_*s*_ = −0.02, *p*_adj_ = 0.896) demonstrated near-zero rank correlations with total lesion volume, whereas Agglomerative clustering exhibited a weak unadjusted trend (*r*_*s*_ = 0.25, *p*_raw_ = 0.016) that likewise failed to survive multiple comparison correction (*p*_adj_ = 0.161). Overall, this lack of strong association suggests that conventional macrostructural lesion burden alone does not serve as a direct surrogate for intralesional microstructural integrity.

We must acknowledge that a significant limitation of the current study is the relatively small sample size of the evaluation cohort (100 subjects), which may restrict the generalizability of our findings. Furthermore, the dataset utilized in this study does not provide patient-level clinical metadata, such as EDSS scores or cognitive assessments. While we have demonstrated that the RLIS metric does not show a significant association with radiological burden, we cannot yet confirm its correlation with pathophysiological features like clinical disability. Furthermore, a key methodological consideration of the current framework is the reliance on the lesion-spared IC-CST as a reference. While the IC-CST provides a highly stable, anisotropic benchmark that reduces the risk of contamination from periventricular NAWM, baseline DTI properties inherently vary across different white matter tract architectures. Consequently, the derived index represents operational microstructural similarity to a high-integrity IC-CST reference rather than tract-specific biological preservation.

A physical limitation of this study is the presence of uncorrected spatial systematic errors (SSEs) associated with spatial variations in the local b-matrix, *b*(**r**). While gradient nonlinearity is one source of spatial variation, *b*(**r**) ultimately reflects the full local diffusion-encoding gradient waveform across the volume according to the generalized Stejskal–Tanner equation (Borkowski and Krzyżak, 2018). Literature indicates that across a typical 25 cm field of view, these uncorrected spatial variations can bias MD estimates by approximately –10% to +20% (potentially reaching up to 30% farther from the isocenter) and induce angular deviations in tensor orientation ranging from 3° to 18° (Bammer et al., 2003; Borkowski and Krzyżak, 2018). Although lesions were distributed across multiple brain regions, this spatial distribution alone does not rule out potential contributions from spatial variations in *b*(**r**) to the observed measurements. Future studies should implement direct spatial *b*-matrix calibration, such as *B*-matrix Spatial Distribution DTI (Borkowski and Krzyżak, 2018) and incorporate voxelwise correction, ideally validated with isotropic phantoms, to distinguish hardware-related SSEs from true microstructural heterogeneity (Hansen et al., 2021). Addressing these hardware-related variations, alongside evaluating test–retest reliability, will be critical for multi-center standardization. Furthermore, future longitudinal studies incorporating patient-level clinical metrics will be necessary to assess the parametric stability of this metric over time and establish its prospective correlation with clinical disability and disease progression.

## 5 Conclusion

In this work, we introduced a robust automated multimodal pipeline designed to segment MS lesions and quantify the operationally defined percentage of reference-like intralesional segments. A key contribution of this study is the development of a modified 2.5D U-Net architecture. By implementing residual blocks, attention gates, and deep supervision, the complete architecture achieved higher nominal sensitivity metrics than the contextual benchmark. These architectural features contributed to a higher nominal Lesion-wise True Positive Rate (LTPR = 0.831 [95% CI: 0.778–0.879], relative to the published benchmark of 0.542) and a strong Volume Correlation (VC = 0.954 [95% CI: 0.914–0.995]), reflecting consistent global lesion load estimation despite precision-related trade-offs.

Regarding the identification of reference-like tissue segments, our analysis suggests that K-Means and GMM are the most empirically stable algorithms for this purpose, offering conservative and consistent estimations across the cohort. Most importantly, our results showed limited or no association between the identified percentage of RLIS and traditional radiological metrics, such as lesion volume and count.

This lack of strong association suggests that the operationally defined RLIS metric provides localized microstructural information that varies distinctly from conventional lesion load. This metric provides a quantitative descriptor of intralesional microstructural similarity to subject-specific lesion-spared tissue. Consequently, it may serve as a candidate imaging metric for future investigation of the clinico-radiological paradox. Although the present study is limited by the absence of patient-level clinical outcomes, it establishes a baseline for future longitudinal research. Such research will be vital to validating this metric as a future tool for supporting clinical outcome prediction, pending validation with patient-level clinical data.

## Data Availability

Publicly available datasets were analyzed in this study. This data can be found here: https://springernature.figshare.com/articles/dataset/MSLesSeg-_baseline_and_benchmarking_of_a_new_Multiple_Sclerosis_Lesion_Segmentation_dataset/27919209/1 https://zenodo.org/records/10931121.
